# Effects of intratesticular injection of hypertonic mannitol and saline on the quality of donkey sperm, indicators of oxidative stress and testicular tissue pathology

**DOI:** 10.1186/s12917-024-03915-1

**Published:** 2024-03-11

**Authors:** Mohammadreza Baqerkhani, Ali Soleimanzadeh, Rahim Mohammadi

**Affiliations:** 1 Department of Theriogenology, Faculty of Veterinary Medicine, Urmia University, P.O. Box: 57561-51818, Urmia, Iran; 2 Department of Surgery and Diagnostic Imaging, Faculty of Veterinary Medicine, Urmia University, Urmia, Iran

**Keywords:** Apoptosis, Pyroptosis, Genes expression, Chemical sterilization, Donkey

## Abstract

**Objectives:**

The aim of the present study was to examine donkey sperm quality after intratesticular injection of hypertonic mannitol (HM) and saline (HS).

**Methods:**

Randomly assigned to five treatment groups were 15 adult male donkeys: (1) Control group (no treatment), (2) Surgery group (surgical castration for testosterone control), (3) NS group (normal saline intratesticular injection), (4) HS group (hypertonic saline), and (5) HM group. We injected 20 mL per testicle. We took 5 mL blood from all donkeys before injection. Castration was performed under general anesthesia 60 days later. Samples included blood and testicular tissue. Total motility (TM), progressive motility (PM), movementy features, DNA damage, morphology, viability, and plasma membrane functionality were evaluated. Hormone analyses, histomorphometric studies and oxidative stress indices including total antioxidant capacity (TAC), glutathione peroxidase (GPx), glutathione (GSH), superoxide dismutase (SOD), malondialdehyde (MDA), and NADP+/NADPH were evaluated. Apoptosis, pyroptosis-related *Bax*, *Caspase-1*, *GSDMD*, and *Bcl-2* expression were also assessed.

**Results:**

In HS and HM groups, testosterone, epididymal sperm count, motility, viability, and plasma membrane functionality dropped while sperm DNA damage increased. HS and HM groups had significantly lower histomorphometric parameters, TAC, GPx, SOD, GSH, and *Bcl-2* gene expression. MDA, NADP^+^/NADPH, *Bax*, *Caspase-1*, and *GSDMD* gene expression were substantially higher in the HS and HM groups than in the control group.

**Conclusions:**

Toxic effects of hypertonic saline and mannitol on reproductive parameters were seen following, hence, they might be considered as a good chemical sterilizing treatment in donkeys.

## Introduction

The primary goal of management of reproduction is to manage and mitigate undesirable male sexual behavior, reduce animal population size and inhibit physiological sexual activity [1]. In recent decades, surgical castrarion as a method of male sterilization has been declined [2]. This shift can be attributed to several factors including the significant costs associated with surgery, the time-consuming nature of the procedure, the need for post-operative care and treatment, the potential risk of post-operative complications and the need for anesthesia and sterilization and the need for well-trained surgeons and appropriate medical equipment. Therefore, alternative options such as chemical castration have received a lot of research attention and are presented as valuable alternatives [1]. Chemical castration has several advantages including relieving discomfort and stress in animals, reducing incidence of infection and bleeding, and the potential for widespread use [3]. This experimental technique involves administration of chemical agents directly into the testes, vas deferens or tail of the epididymis with the goal of inducing genital cell degeneration [3, 4].

Chemical castration is the suppression of sperm production in the testicles by introducing a chemostatic substance into either the testicles or the epididymis. This chemical induces sclerosis of cells and tissues, thereby inhibiting sperm formation [5]. One of the main concerns is the harmful side effects of the chemicals [6]. Different researchers have used different methods of chemical sterilization on different species of animals, with varying results [2, 7]. Several active ingredients have been used in animal studies including ethanol, chlorhexidine, formaldehyde, glycerol, acetic acid, lactic acid, sodium chloride, calcium chloride, glucose, zinc-containing compounds, normal saline, HM, and hypertonic saline. These studies were conducted on a variety of animal species including rats, stallions, cats, donkeys, calves, rams, wild boar and squirrel monkeys. In several cases their effectiveness was evident, however, not in others. The toxic substances to genital cells have been shown to activate the mechanisms of necroptosis and apoptosis leading to cell death and elimination of genital cells [1, 6, 8–18].

The study by Kanter et al. (2013) provided empirical evidence supporting the claim that oxidative stress has deleterious effects on germ cells in the testis of rats and ultimately leads to the occurrence of apoptosis in these cells [19]. Apoptosis is a crucial mechanism of programmed cell death as it plays a critical role in maintaining tissue homeostasis, facilitating embryonic development and supporting immune responses. Significantly, the deregulation of apoptosis plays a crucial role in the development of tumors, neurological diseases and autoimmune diseases [20]. Apoptosis and pyroptosis have several characteristics, such as DNA damage and chromatin condensation [21, 22]. Nevertheless, the distinct morphological features of pyroptosis are obviously different from those of apoptosis. Apoptosis is generally considered to be a controlled and non-inflammatory mode of cell death. In contrast, pyroptosis triggered by extacellular and intracellular stimuli, including exposure to bacteria, viruses, toxins, and chemotherapeutic agents, can trigger pyroptosis [23]. Within the canonical pathway, inflammasome (a group of *intracellular* multimeric protein complexes that *activate* inflammatory *caspase*-*1*) and caspase-1 activation occurs through the stimulation of intracellular signaling molecules by pathogen-associated molecular patterns (PAMPs) and danger-associated molecular patterns (DAMPs). This process involves assembly of pro-caspase-1 and apoptosis-associated Speck-like protein containing a CARD (ASC). The cleavage process by Caspase-1 involves the cleavage of gasdermin D (GSDMD) as well as Pro-IL-1/18. The N-GSDMD protein induces perforation of the cell membrane through the formation of nonselective holes leading to water infiltration, cell lysis and subsequent cell death in the pyroptosis process. On the other hand, *Bcl-2* family members play a crucial role in the apoptosis process in regulating apoptosis [24] via modulating the permeability of the mitochondrial outer membrane and initiating the process of programmed cell death. The family in question consists of both anti-apoptotic (*Bcl-2*) and pro-apoptotic (*Bax*) members. *Bax* and *Bcl-2* have been identified as potential regulators of germ cell apoptosis as documented in previous studies [25, 26].

Commonly used formulations include solutions of sodium chloride (NaCl) at concentrations of 2%, 3%, 5%, 7%, and 23%. For prevention and treatment of secondary brain damage, the hypertonic saline (HS) is used. The primary mechanism of action is an osmotic effect, but other effects are sometimes observed [27]. HS has an effect on osmosis. Plasma osmolarity increases after intravenous HS administration. Administration of hypertonic saline can induce acute local osmotic shock and result in widespread necrosis. Also, this approach was effective in sterilization in adult canine and rat models [28, 29].

HM, a sugar alcohol, is incompletely metabolized and is excreted in its native form through the urinary tract. Maadi et al. (2021) demonstrated that single intratesticular injection of HM resulted in sterilizations in rats [29].

It has been observed that some young male donkeys may exhibit unwanted sexual behavior at the age of five to six months. This behavior can cause distress to the animal’s dam or other animals kept with him, and it can establish patterns that are difficult to change even after castration [30]. Given that castration through surgery is riskier in donkeys than in horses or ponies, we hypothesized that HM and HS castration could be a more suitable alternative. Based on a risk/benefit analysis and welfare concerns, HM and HS castration may be a better option. There have been no previous reports of intratesticular HM and HS injections in male donkeys, so this study aimed to evaluate the effects of a single intratesticular injection of HM and HS in donkeys.

## Materials and methods

### Ethics statement

Under the approved protocol number IR-UU-2382/PD/3, management techniques and care protocols were carried out in accordance with the rules of Urmia University’s Animal Ethics Committee, located in Urmia, Iran.

### Studied animals

Fifteen adult male donkeys weighing 120–150 kg and aged 2–2.5 years were included in this study. The animals were fed with alfalfa hay with unlimited access to water. All animals had a two-week acclimatization period before testing to reduce the deleterious effects of unfamiliar stress on test results. The donkey’s libido was also observed during the study period. It should be noted that all animals were released after the treatment period.

### Experimental protocol

**Fig. 1:**
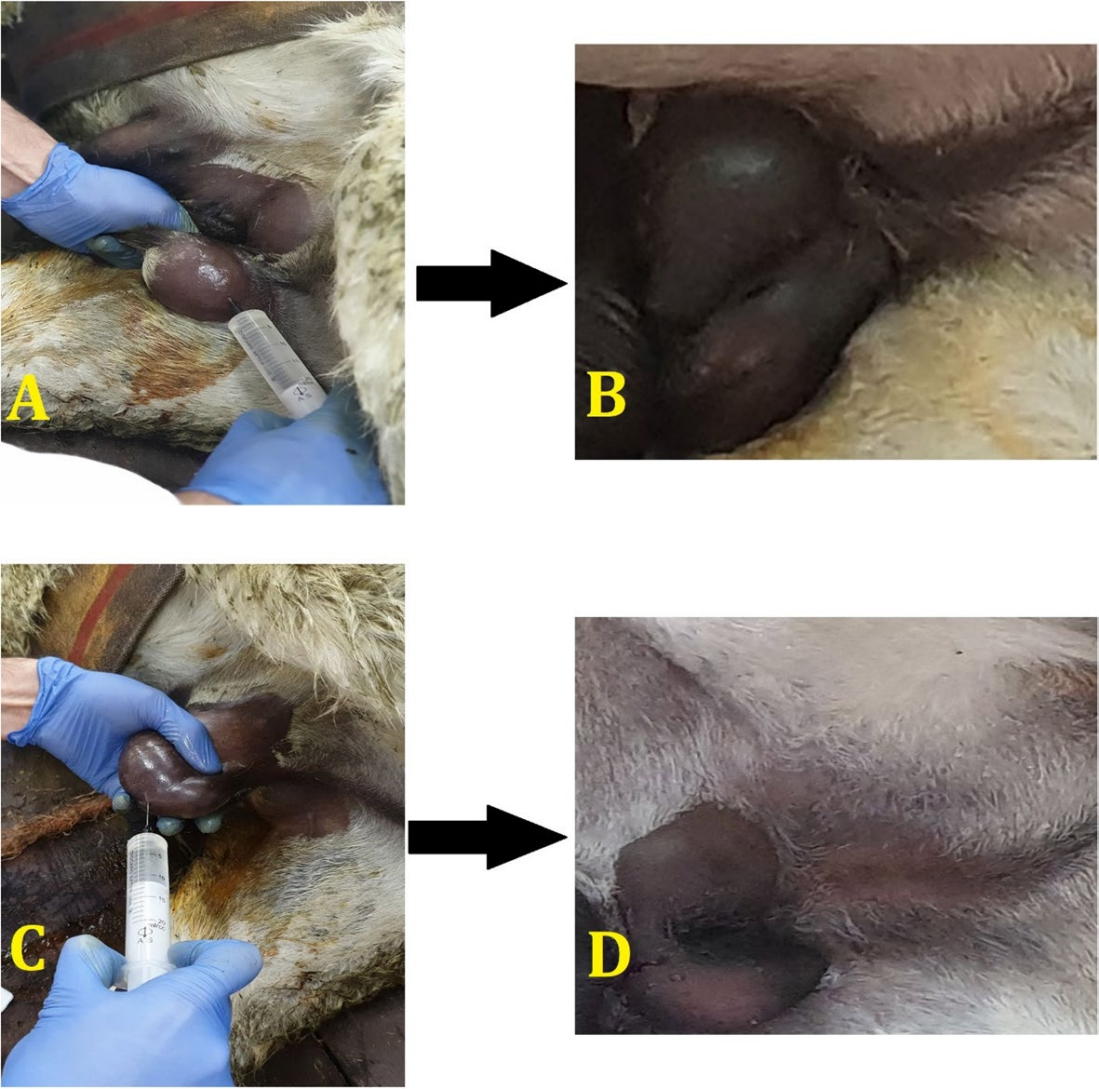
**A **Intratesticular injection of normal saline, (**B**) Day 60 of normal saline group, (**C**) Intratesticular injection of mannitol, (**D**) Day 60 of mannitol group

Fifteen adult male donkeys were randomly assigned to one of five treatment groups: (1) Control group (no treatment), (2) Surgery group (surgical castration for testosterone control), (3) NS group (receiving a single bilateral intratesticular injection (20 mL per testis) of normal saline (sodium chloride 0.9%, Iran Injection and Pharmaceutical Products Company, Tehran, Iran), (4) HS group (receiving a single bilateral intratesticular injection of sodium chloride 20%, Iran Injection and Pharmaceutical Products Company, Tehran, Iran), and (5) HM group (receiving a single bilateral intratesticular injection of HM 20% (Iran injection and pharmaceutical products company, Tehran, Iran) (Fig. 1), [3, 29].

### Intratesticular injection

The animals were physically restrained and given moderately safe general anesthesia using acepromazine (Neurotranq PRO INJ. Alfasan, Woerden, The Netherlands) 0.05 mg/kg body weight and ketamine (Ketamine 10% PRO INJ. Alfasan, Woerden, The Netherlands) 2 mg/kg B.W. The animals were then placed in a lateral position. Following surgical prep, the injection started with the upper testicle. The site of injection was the caudal pole of the testicle, which was near the tail of the epididymis. A 27 gauge needle was passed from the caudoventral side of each testis towards the dorsocranial side of the testicle in a completely aseptic environment. As the needle was withdrawn from the proximal to the distal end, the solution was carefully deposited throughout the course by linear infiltration. Each donkey in the HM, hypertonic saline and normal saline groups received the above treatment [12]. Care was taken to prevent leakage of the solution from the injection site. Injection into the testicles was classified as a zero day.

After recovery, all donkeys were kept until day 60 [4]. The donkeys received intramuscularly procaine penicillin and dihydrostreptomycin sulfate at a dose of 1 mL/kg body weight (Pen & Strep, 1 mL contains 200 mg procaine penicillin and 250 mg dihydrostreptomycin sulfate, Norbrook Co., Ireland). Phenylbutazone at a dose of 1.1 mL/50 kg body weight (butadion, 1 mL contains 200 mg phenylbutazone, NASR FARIMAN Co., Iran) was administered for three consecutive days after each procedure [31]. The libido was monitored [32] for an additional 60 days. Animals of all groups were surgically castrated after 60 days.

### Scrotal circumference and testicular volume determination

The circumference of the scrotum was measured with a measuring tape before castration. An ellipsoidal equation was used to calculate testis length, width, height, and volume (0.523 × height × length × width) [33].

### Blood sampling

For all five groups, jugular veins were taken for blood samples on 0 and 60 days after the start of the experiments in a completely sterile environment [3]. For serum isolation, the blood tubes were centrifuged at 2000×*g* for 5 min. The serum samples were separated and kept at – 20 °C until analyzed to detect testosterone levels.

### Serum testosterone assay

An enzyme-linked immunosorbent assay kit (Monobind Inc., Lake Forest, USA) was used to determine testosterone levels according to the manufacturer’s instructions.

### Collection of epididymal sperm

The cauda epididymis was removed from the testis after surgical castration. It was then added to Tyrode’s medium containing the following components: 96 mm NaCl, 3.1 mm KCl, 2 mm CaCl2, 0.4 mm MgSO4, 0.3 mm KH2PO4, 50 g kanamycin/mL, 20 mm HEPES, 5 mm glucose, 21.7 mm sodium lactate, and 1 mm sodium pyruvate [34]. Multiple incisions were made in the tail of the epididymis to enhance sperm extraction. The culture medium was then placed in a CO_2_ incubator for 2 h and the released sperm were then used in the culture medium to test various parameters [35].

### Sperm count

To count the sperm, a dilution of 1:20 of the sperm was carried out. A 1 mL microtube was first filled with 190 µL of distilled water and then with 10 µL of the required semen. Then 10 µL of this mixture was poured down a Neobar slide (which was already covered with a rock slide). 200 sperm were analyzed under 400× magnification in each sample on the Neobar slide and the results were expressed as a percentage [36].

### Motility and motion parameters

A CASA system (Test Sperm 3.2; Videotest, St. Petersburg, Russia) was used to measure total motility (%), progressive motility (%), curvilinear velocity (VCL; µm s^–1^), straight-line velocity (VSL; µm s^–1^), average path velocity (VAP; µm s^–1^), straightness (STR; %), linearity (LIN; %) amplitude of lateral head displacement (ALH; µm s^–1^), and beat-cross frequency (BCF; Hz) (Table 1; [37]). Ten microliters of semen were used to evaluate at least 500 spermatozoa in 5 microscopic fields [38].

**Table 1:** Parameter settings for the CASA

**Parameter**	**Setting**
**Frame rate**	60 frames/sec (Hz)
**Per field**	45 frames
**Duration of capture**	1 s
**Stage temperature rate**	37 °C
**Sperm recognition**	15–75 μm^2^
**Chamber type**	Slide-Coverslip (22*22 mm)
**Volume per slide**	7 μL
**Chamber depth**	≈ 20 μm
**Minimum number of field analysis**	500 cells
**sample dilution**	20 × 10^6^
**Image type**	Phased contrast
**Progressive spermatozoa**	VAP ≥ 75 μm/s and STR ≥ 80%

### Evaluation of sperm viability (plasma membrane integrity) and morphology

According to a WHO procedure, eosin-nigrosine staining was used to assess viability and morphology [39]. Eosin and nigrosine dyes (Merck, Darmstadt, Germany) were prepared in distilled water. One volume of semen was mixed with two volumes of 1% eosin, and the mixture was then examined at 400× magnification using a light microscope (model CHT, Olympus Optics Co. Ltd.). Live spermatozoa were white and transparent on the head since they did not absorb color, while the dead spermatozoa absorbed a red-purple color [40–46] Eosin-nigrosine staining was also performed to analyze the percentage of abnormal sperm. Sperm that had parts of their head, neck, or tail discolored were considered dead sperm. In this sense, sperm showing cytoplasmic remnants as well as other obvious defects were classified as abnormal sperm. For each sample, 200 sperm were examined at a magnification of 400× and the results presented as a percentage [38].

### DNA damage evaluation

To examine DNA damage and identify denatured, double-stranded DNA fragments in sperm chromatin, acridine orange (AO) staining was used [47]. Carnoy’s fixative, 1:3 ratio of methanol and acetic acid, was used to fix a thick smear for 2 h before it was removed and allowed to air dry at room temperature for 5 min. The smear was then immersed in a stock solution of 1 mg acridine orange and 1000 mL distilled water, which was then kept in the dark at 4 °C for 5 min [48]. Sperm that fluoresced yellow or red were thought to be damaged. The sperm were examined with a fluorescence microscope (Model GS7, Nikon Co., Tokyo, Japan) emitting light with a wavelength of 490 nm.

### Sperm plasma membrane functionality (PMF)

Hypoosmotic Swelling Test (HOST) involves diluting 10 µL of semen in 100 µL of a hypoosmotic solution containing 1.35 g fructose and 0.73 g sodium citrate and incubating for 30 min at 37 °C [40, 49]. After incubation, sperm are checked for sperm PMF using a contrast phase microscope (Olympus, BX41, Tokyo, Japan) at 400× magnification. To determine the proportion of sperm with an intact plasma membrane, 200 straight or curved sperm are usually counted.

### Assessment of enzymatic antioxidant activity

For this purpose, 20–30 mg of testicular tissue were homogenized in 1000 µL of lysis buffer (150 mM sodium chloride, 50 mM Tris-HCl, pH 7.4, 1 mM ethylenediaminetetraacetic acid, 1 mM phenylmethylsulfonyl fluoride, 1% Triton X-100, 1% sodium deoxycholic acid, 0.1% sodium dodecylsulfate, 5 µg/mL of aprotinin, 5 µg/mL of leupeptin), centrifuged (9000 rpm, 15 min) and the supernatant was collected for biochemical investigations [50]. Also, the method of Lowry et al. (1951) [51] was used to determine the protein levels.

### Determination of total antioxidant capacity (TAC)

A TAC kit (Naxifer™; Navand Salamat Company, Urmia, Iran) was used to quantify of TAC in testis [38]. The TAC values ​​were given in nmol/mg protein.

### Glutathione peroxidase activity

A GPx kit (Nagpix™, Navand Salamat Company, Urmia, Iran) was used to calculate the GPx level in testis [38]. The GPx value was given in mU/g protein.

### Superoxide dismutase evaluation

The amount of Superoxide dismutase (SOD) in the testis sample was determined using a SOD test kit (Nasdox™, Navand Salamat Company, Urmia, Iran). To measure the SOD activity, the decrease in color development at 405 nm, which is regarded as an inhibitory effect, was utilized [38]. The SOD activity in testis was expressed in U/g protein.

### Glutathione evaluation

A commercially available GSH kit was used to determine the glutathione level (GSH) in the testis sample. This was performed with the NarGul™ Glutathione Assay Kit-GSH (Navand Salamat Company, Urmia, Iran) [38]. The GSH activity was expressed in µmol/mg protein.

### Amounts of malondialdehyde

Oxidative stress was assessed using an MDA test kit (The Nalondi™ Lipid Peroxidation Assay Kit (NID), Navand Salamat, Urmia, Iran). A spectrophotometer (Thermo Fisher Scientific; Waltham, MA) at wavelength of 523 nm was used to measure the MDA concentration [38]. A standard curve was used to calculate the amount of MDA present, and the concentration was given in nmol/g protein.

### NADP^+^/NADPH evaluation

NADP^+^/NADPH kit (NADP/NADPH Assay Kit-WST, DOJINDO LABORATORIES, Kumamoto, JAPAN) was used to assess testicular NADP^+^/NADPH values. NADP and NADPH levels were determined according to the manufacturer’s instructions. The test was based on glucose dehydrogenase cycle reaction and performed in 180 µL of reaction buffer containing 50 µL of supernatant, 120 µL of master reaction mix (98 L of NADP cycling buffer + 2 L of NADP cycling enzyme mix) and 10 µL of NADPH developer. After 1 h at room temperature, the concentrations of NADP^+^ and NADPH were measured at 450 nm. NADP^+^/NADPH content was assessed based on the formula (Ratio = (NADPtotal - NADPH)/NADP) derived from standard ODs for each sample.

#### Histological examinations and histomorphometric study of testicular tissue

All donkeys were surgically castrated 60 days after intratesticular injection and the right testes of each donkey were used for histological analysis. Gross examination of the testicles after they have been sectioned transversely. After the tunica albuginea was removed, the specimens were cut into 1 cm squares and dried in a graded alcohol series before being immersed in 10% neutral buffer formalin for 7 days. Histopathological sections of four microns thickness were cleared in methyl benzoate, embedded in paraffin wax and then cleared in methyl benzoate. Hematoxylin-eosin was used to stain these sections, which were then viewed under a light microscope. Testicular histological lesions were rated based on Ibrahim et al. [3].

In the histomorphometric examination of the testicular tissue, after the creation of histological sections, the spermatogenesis was determined with the semi-quantitative method of Johnsen’s score (TBS) in 100 seminiferous tubules of each cross section at the same magnification and summed up mean Johnsen’s score (Table 2) and quantitative method in which 200 seminiferous tubules were examined under light microscopy (CHT model, Olympus Optical Co. Ltd., Tokyo, Japan; magnification x200).

**Table 2:** Johnsen scoring system for evaluating testicular damage

**Johnsen score**	**Description of histological criteria**
10	Full spermatogenesis
9	Slightly impaired spermatogenesis, many late spermatids, disorganized epithelium
8	Less than five spermatozoa per tubule, few late spermatids
7	No spermatozoa, no late spermatids, many early spermatids
6	No spermatozoa, no late spermatids, few early spermatids
5	No spermatozoa or spermatids, many spermatocytes
4	No spermatozoa or spermatids, few spermatocytes
3	Spermatogonia only
2	No germinal cells, Sertoli cells only
1	No seminiferous epithelium

To measure seminiferous tubule diameter (STD), 200 round or nearly round seminiferous tubule cross-sections from each animal were examined randomly (one hundred per testis). Then, two vertical diameters of each cross section of the seminiferous tubules were measured using a light microscopy ocular micrometer (Model CHT, Olympus Optical Co. Ltd., Tokyo, Japan) and their mean values ​​were determined [52]. Sixty seminiferous tubules per group were randomized to determine the Sertoli cell index (SCI), repopulation index (RI) and miotic index (MI). SCI is the ratio of Sertoli cells with a distinct nucleus and nucleolus, present in all seminiferous tubules, to the number of germ cells [53]. RI measures the proportion of germ cell-populated tubules that appear to have reached mid-spermatogonial stage or later [54]. To calculate the proportion of cells lost during cell division, the MI (the number of round spermatids for each pachytene primary spermatocyte) was calculated [54]. Calibrated ocular micrometers were used to determine Leydig nuclear diameter (LCND) and Sertoli nuclear diameter (SCND) as reported by Elias and Hyde [55]. To calculate the Tubule Differentiation Index (TDI) and the spermiation index (SPI), 200 transverse sections of the seminiferous tubules were examined at random in each animal (100 per testis). The proportion of seminiferous tubules with at least three developed germ cells was referred to as TDI [56]. The SPI measures the proportion of seminiferous tubules that normally contain sperm [57].

### RNA extraction, cDNA synthesis and qRT-PCR of testicular tissue

We used SinaSyber Blue HF-qPCR Mix (CinnaGen, Tehran, Iran) on a StepOne Real-Time PCR System (Applied Biosystems, USA) with 25 µL reaction system and with real-time semiquantitative polymerase chain reaction technique (qRT-PCR). The cDNA samples were used to measure the relative expression of the *Bax*, *Caspase-1*, *GSDMD* and *Bcl-2* genes by Real-Time PCR using StepOne (Real-Time PCR System, Applied Biosystems) compared to the *GAPDH* gene as a reference gene analyze. The primers used for *Bax*, *Caspase-1*, *GSDMD* and *Bcl-2* are mentioned in Table 3 [59, 60]. The qPCR thermal cycle settings were as follow: A defatting cycle at 95 °C for 10 min, 45 three-step cycles each with 10 s defatting at 95 °C, 30 s compounding at 60 °C and the stretching step at 72 °C for 30 s. Gene expression in the samples was used to calculate relative expression using the following formula: Relative Expression = 2^- (SΔct-CΔct)^, where SΔct is derived by subtracting ct of the reference gene from ct of the tested gene and the CΔct values ​​of ct are internal control samples. To generate the normal distribution of the relative expression values, these numbers were fitted by logarithm of 10 and then examined [61].

**Table 3:** Nucleotide sequences and product size of primers used in reverse transcription-polymerase chain reaction

**Gene name**	**Primer**	**Band size**	**References**
***Bax***	Forward: 5- GAGCTGGACAGTAACATGGAG-3	147 bp	[59]
	Reverse: 5- GGCAAAGTAGAAAAGGGCAAC-3		
***Bcl-2***	Forward: 5- CCTGTGGATGACTGAATACCTG-3	120 bp	[59]
	Reverse: 5- CAGGAGAAATCAAACAGCGG -3		
***Caspase-1***	Forward: 5- GGGCACGGGTACAGTAAATAG-3	114 bp	[60]
	Forward: 5- CGGGCCTTATCCATAACTGTAG-3		
***GSDMD***	Forward: 5- GTTATTGGCTCTGACTGGGAC-3	148 bp	[60]
	Forward: 5- TGAATCCTGACACGCTCTTG-3		
***GAPDH***	Forward: 5- GAAAGCTGCCAAATACGATGAG- 3	136 bp	[59]
	Reverse: 5- GAAGGTGGAAGAGTGGATGTC- 3		

### Statistical analysis

The data collected in the study were analyzed using SPSS software (version 26.0, IBM Corporation, Chicago, USA). One-way ANOVA was used to assess whether there were significant differences between the groups being compared. Tukey’s post hoc analysis was performed to identify which specific groups differed significantly. Statistical significance was defined as a *p*-value of ≤ 0.05.

## Results

### Clinical findings

No side effects such as scrotal irritation, fistula formation or discharge were observed after intratesticular injections. Slight scrotal edema was noted due to the injections that was decreased by the end of the first week. Self-mutilation due to testicular pain was not noted in any of the donkeys after the intratesticular injections and probing of the scrotum were well tolerated, with no signs of discomfort or an aggressive response. No problems were found in surgically castrated donkeys.

### Plasma testosterone concentration

Table 4 shows the results of serum testosterone levels in different experimental groups. The serum testosterone level of donkeys in the HM and HS groups showed a significant drop at day 60 compared to day 0. The findings showed that testosterone levels were lower in the HM and HTS groups compared to the control group (*p* ≤ 0.05, Table 4). On the other hand, there was no significant difference between the control group and the normal saline group (*p* ≤ 0.05, Table 4).

**Table 4:** Effect of intratesticular injection on serum testosterone concentration in groups at day 0 (pre-castration) compared to day 60. Values are expressed as means ± SEM

**Groups**	**Control**	**Surgery**	**NS**	**HM**	**HS**
**Analysis**					
**Testosterone (ng/ml) on day 0**	6.74 ± 0.45^a^	6.53 ± 0.31^a^	6.69 ± 0.38^a^	6.57 ± 0.29^a^	6.63 ± 0.36^a^
**Testosterone (ng/ml) on day 60**	6.81 ± 0.24^a^	0.45 ± 0.17^c^	6.42 ± 0.31^a^	1.09 ± 0.11^b^	1.37 ± 0.19^b^

*NS* normal saline, *HM* hypertonic mannitol, *HS* hypertonic saline

Different superscripts within the same row demonstrate significant differences (*p* ≤ 0.05)

### Concentration, motility of the sperm and motility characteristics

Table 5 shows the semen concentrations measured in all test groups. These results showed that the semen concentration of the control and normal saline groups was significantly higher than that of the HM and HS groups (*p* ≤ 0.05; Table 5).

**Table 5:** Effects of intratesticular injections on epididymal sperm concentration, total and progressive motility, and motility characteristics of donkey testicles in different experimental groups. Values are expressed as mean ± SEM

**Groups**	**Control**	**NS**	**HM**	**HS**
**Analysis**				
**Epididymal sperm concentration (10**^**6**^**/mL)**	8247.91 ± 475.9^a^	8195.50 ± 236.7^a^	981.25 ± 135.1^b^	1045.39 ± 207.6^b^
**Total motility (%)**	85.83 ± 2.43^a^	82.17 ± 2.91^a^	29.05 ± 1.77^c^	34.81 ± 1.65^b^
**Progressive motility (%)**	31.25 ± 1.01^a^	29.50 ± 1.16^a^	7.51 ± 0.81^b^	7.64 ± 0.37^b^
**VAP (μm/s)**	129.03 ± 5.75^a^	127.69 ± 3.90^a^	61.58 ± 1.22^c^	67.23 ± 1.05^b^
**VCL (μm/s)**	205.67 ± 8.14^a^	194.23 ± 9.47^a^	94.86 ± 3.92^b^	97.49 ± 3.55^b^
**VSL (μm/s)**	115.41 ± 3.35^a^	109.92 ± 4.81^a^	70.44 ± 2.40^c^	83.30 ± 2.98^b^
**LIN (%)**	42.25 ± 1.20^a^	41.49 ± 1.29^a^	30.61 ± 1.17^b^	32.19 ± 1.11^b^
**ALH (μm/s)**	2.69 ± 0.18^a^	2.60 ± 0.22^a^	1.45 ± 0.16^b^	1.49 ± 0.17^b^
**STR (%)**	81.78 ± 2.70^a^	78.20 ± 2.31^a^	52.71 ± 1.45^b^	54.67 ± 2.61^b^
**BCF (Hz)**	34.58 ± 1.15^a^	32.41 ± 1.27^a^	23.57 ± 0.83^b^	23.19 ± 1.19^b^

*NS* normal saline, *HM* hypertonic mannitol, *HS* hypertonic saline, *VAP* Average Path Velocity, *VCL* Curvilinear Velocity, *VSL* Straight-Line Velocity, *LIN* Linearity, *ALH* Amplitude of Lateral Head displacement, *STR* Straightness, *BCF* Beat-Cross Frequency

Different superscripts within the same row demonstrate significant differences (*p* ≤ 0.05)

Groups HM and HS in terms of sperm total and progressive motility provided poor outcomes compared to the control group (*p* ≤ 0.05; Table 5). Furthermore, there were no significant differences in the control and normal saline groups (*p* > 0.05; Table 5).

Table 5 shows the characteristics of donkey sperm motility rates. The groups receiving HM and HS showed decreased VCL, VSL, VAP, LIN, STR and BCF compared to the control group (*p* ≤ 0.05; Table 5). However, injection of normal saline into the testicles of donkeys had no significant effects compared to the control group (*p* ≤ 0.05, Table 5).

### Plasma membrane functionally, DNA damage sperm, viability and sperm abnormalities

**Fig. 2:**
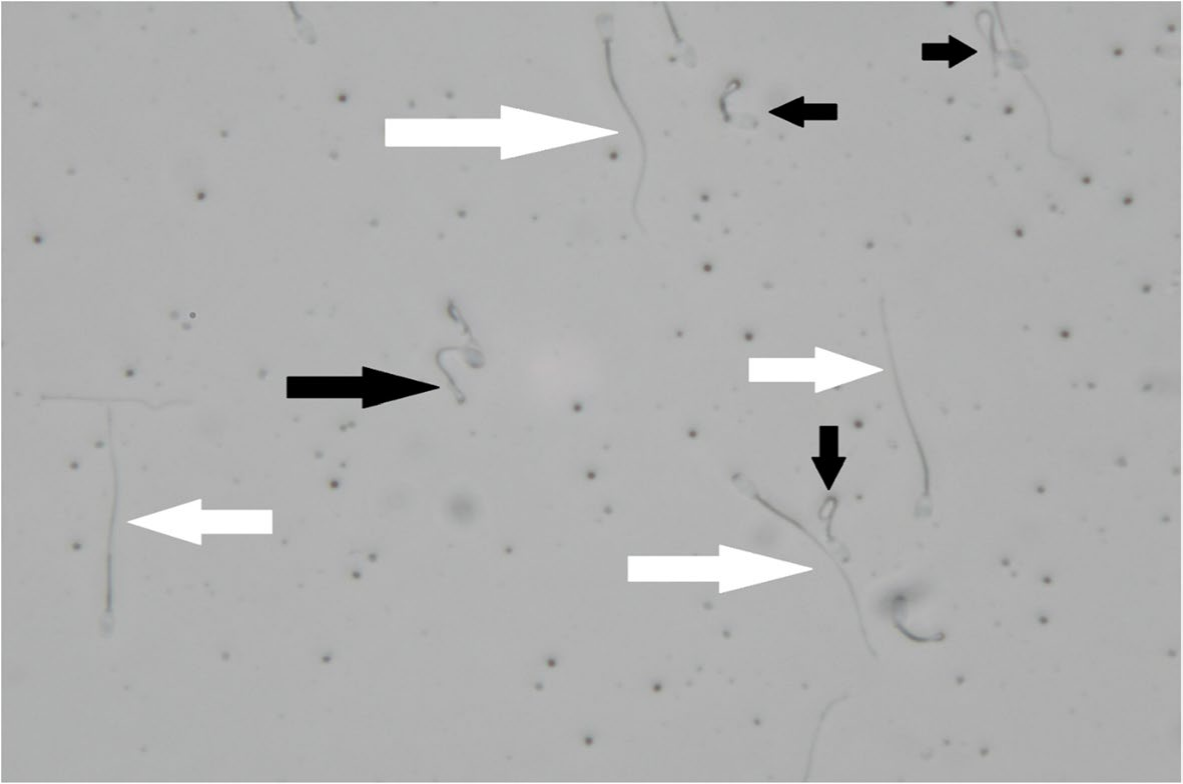
Sperm PM functionality. White arrows - the spermatozoa with straight tails (nonfunctional PM); Black arrows - the spermatozoa with coiled tails (functional PM). (400 ×)

**Fig. 3:**
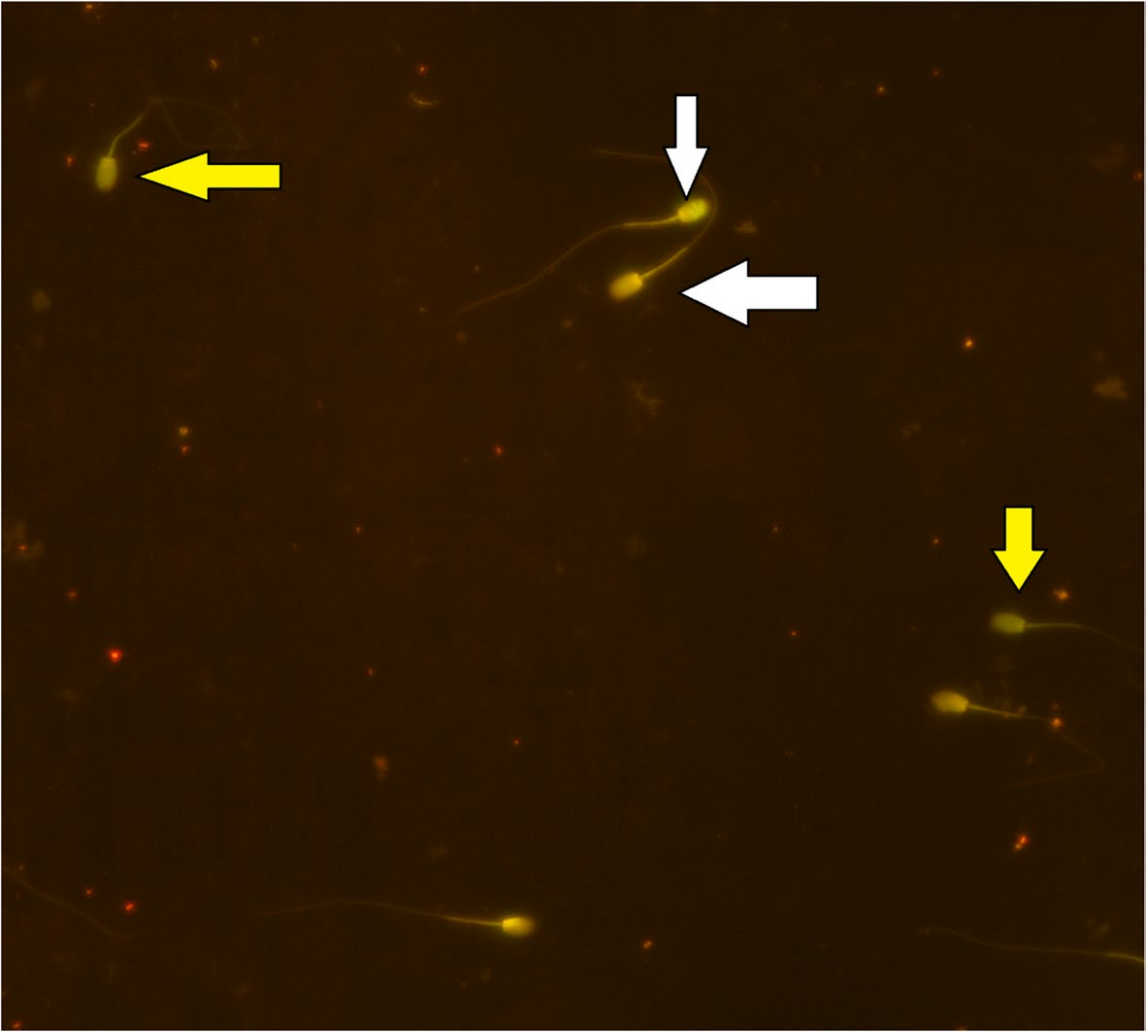
Sperm DNA damage. Yellow arrows - normal spermatozoa (green); White arrows - DNA damaged spermatozoa (yellow-red). (Acridine orange, 400×)

**Fig. 4:**
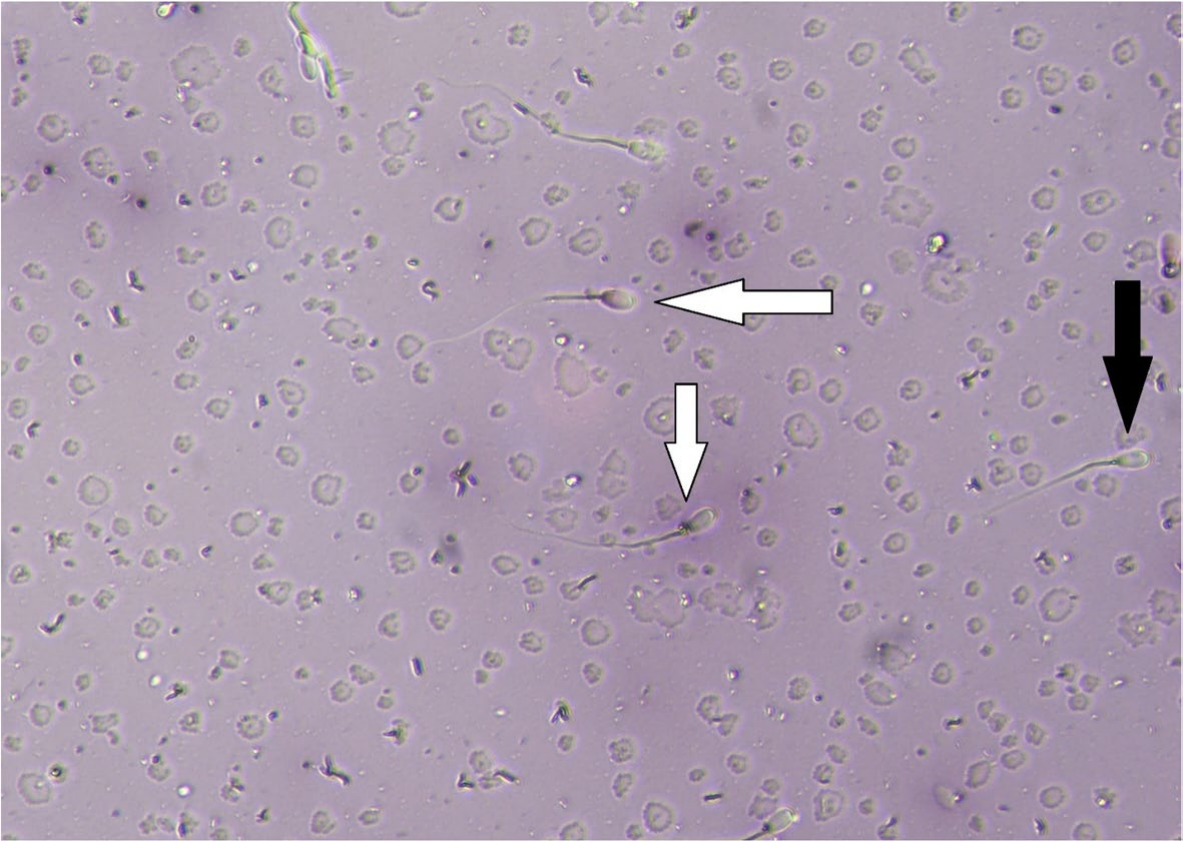
Sperm viability. Black arrow - Viable spermatozoa (colorless); White arrows - Dead spermatozoa (red). (Eosin/nigrosine, 400×)

The HM and HS groups had more sperm with damaged PMF compared to the other groups. In addition, the PMF results showed that there were no significant differences between the normal saline group and the control group (*p* ≤ 0.05, Table 6; Fig. 2). Table 6 shows the DNA integrity of donkey sperm. Compared to the control group, injection HM and HS reduced sperm DNA integrity (*p* ≤ 0.05, Fig. 3), and there were also no significant differences between the control group and the normal saline group (*p* > 0.05). In addition, we found that sperm viability was lowest in the HM and HS groups compared to the control group (*p* ≤ 0.05, Fig. 4). However, there were no significant differences in viability percentage between the other groups (*p* > 0.05). Table 6 shows the percentage of sperm abnormalities measured in all experimental groups. The HM and HS groups had a significantly higher number of head and tail abnormalities compared to the other groups (*p* ≤ 0.05). In addition, the proximal and distal droplet percentages were higher in the HM group than other groups (*p* ≤ 0.05, Table 6).

**Table 6:** Effects of intratesticular injections on plasma membrane functionality (PMF), DNA damage, viability and abnormal morphology of donkey testicles in different experimental groups. Values are expressed as mean ± SEM

Groups		Control	NS	HM	HS
Analysis					
**Sperm plasma membrane functionality (%)**		90.68 ± 3.22^a^	86.65 ± 1.93^b^	39.87 ± 1.26^d^	46.19 ± 1.74^c^
**DNA damage (%)**		1.73 ± 0.20^b^	2.08 ± 0.17^b^	18.93 ± 0.90^a^	17.75 ± 0.87^a^
**Viability (%)**		89.19 ± 2.60^a^	86.68 ± 3.32^a^	37.19 ± 1.61^c^	41.22 ± 1.19^b^
**Abnormalities sperm morphology %**	**Head abnormality %**	8.22 ± 1.19^b^	7.47 ± 0.78^b^	29.36 ± 1.19^a^	31.80 ± 1.45^a^
	**Tail abnormality %**	4.15 ± 0.58^c^	3.80 ± 0.41^c^	16.62 ± 1.43^b^	20.38 ± 0.61^a^
	**Proximal droplet %**	12.31 ± 1.24^b^	10.19 ± 1.21^b^	23.08 ± 1.21^a^	21.35 ± 1.77^a^
	**Distal droplet %**	11.79 ± 1.44^b^	9.73 ± 1.86^b^	31.82 ± 1.35^a^	28.79 ± 1.30^a^
	**Normal %**	68.42 ± 2.71^b^	65.80 ± 1.08^b^	21.63 ± 0.71^a^	20.77 ± 0.56^a^

*NS* normal saline, *HM* hypertonic mannitol, *HS* hypertonic saline

^a,b,c,d^Different superscripts within the same row demonstrate significant differences (*p* ≤ 0.05)

#### Analysis of testicular antioxidant activities

**Fig. 5:**
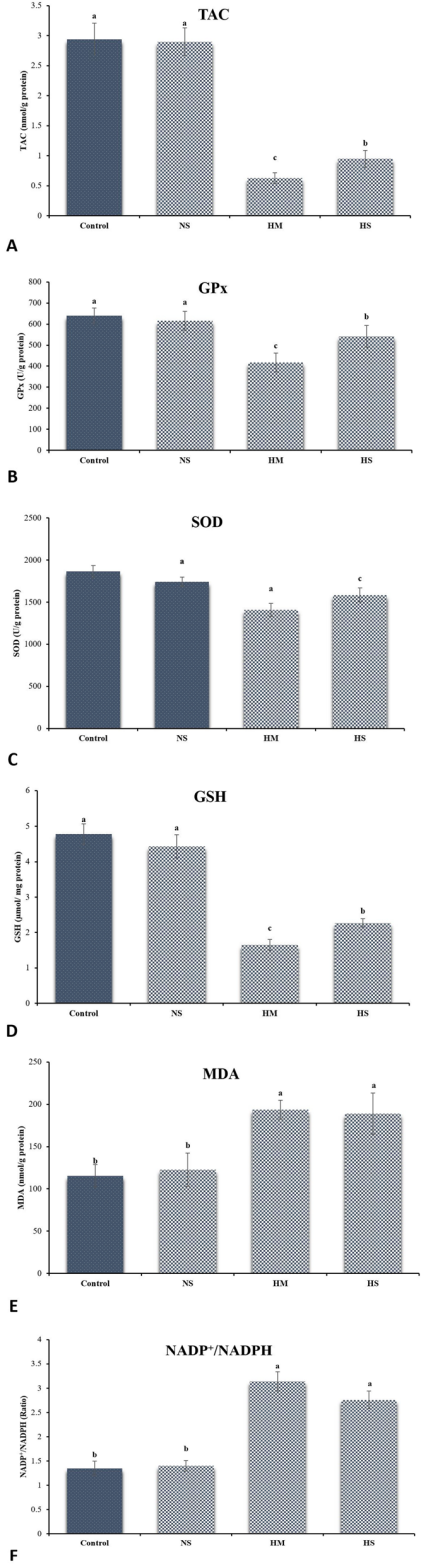
**A **Total antioxidant capacity (TAC); (**B**) Glutathione peroxidase (GPx); (**C**) Superoxide dismutase (SOD); (**D**) Glutathione (GSH) activities; (**E**) Lipid peroxidation (MDA); (**F**) NADP^+^/NADPH of donkey testes after intratesticular injection of hypertonic mannitol and hypertonic saline (HTS) in different experimental groups. NS: normal saline; HM: hypertonic mannitol; HS: hypertonic saline. ^ab^Different superscripts within the same row demonstrate significant differences (*p* ≤ 0.05; Mean ± S.E.M.)

To analyze the effect of injected HM on testicular markers of oxidative stress in donkeys, TAC, GPx, SOD, GSH, MDA and NADP^+^/NADPH levels were evaluated (Fig. 5). The HM and HS groups showed significantly lower TAC, GPx, SOD and GSH levels compared to the control group (*p* ≤ 0.05, Fig. 5A, B, C, D), At the same time, there was no significant difference between normal saline and the control group (*p* > 0.05; Fig. 4A, B, C, D). Analysis of MDA and NADP^+^/NADPH levels showed a significant increase in the HM and HS group compared to the other group (*p* ≤ 0.05, Fig. 4E, F). Notably, normal saline injection had no effect on MDA and NADP^+^/NADPH levels compared to the control group (*p* > 0.05; Fig. 5E, F).

#### Testicular histomorphometric study and histopathology

HM and HS led to drastic morphological changes in the testes Atrophied seminiferous tubules showed severe hypocellularity (reduction in the number of germ cells) and intraepithelial vacuolation. Cracks, vacuolation, vascular occlusion, inflammatory cell infiltration, accumulation of edematous fluid and dilation of the interstitial space were also observed in the intratubular connective tissue. In these samples, Leydig cells were degenerated and showed pyknotic nuclei. In addition, Sertoli cells lost their connection with germ cells and appeared amorphous, irregular and smaller nuclei.

**Fig. 6:**
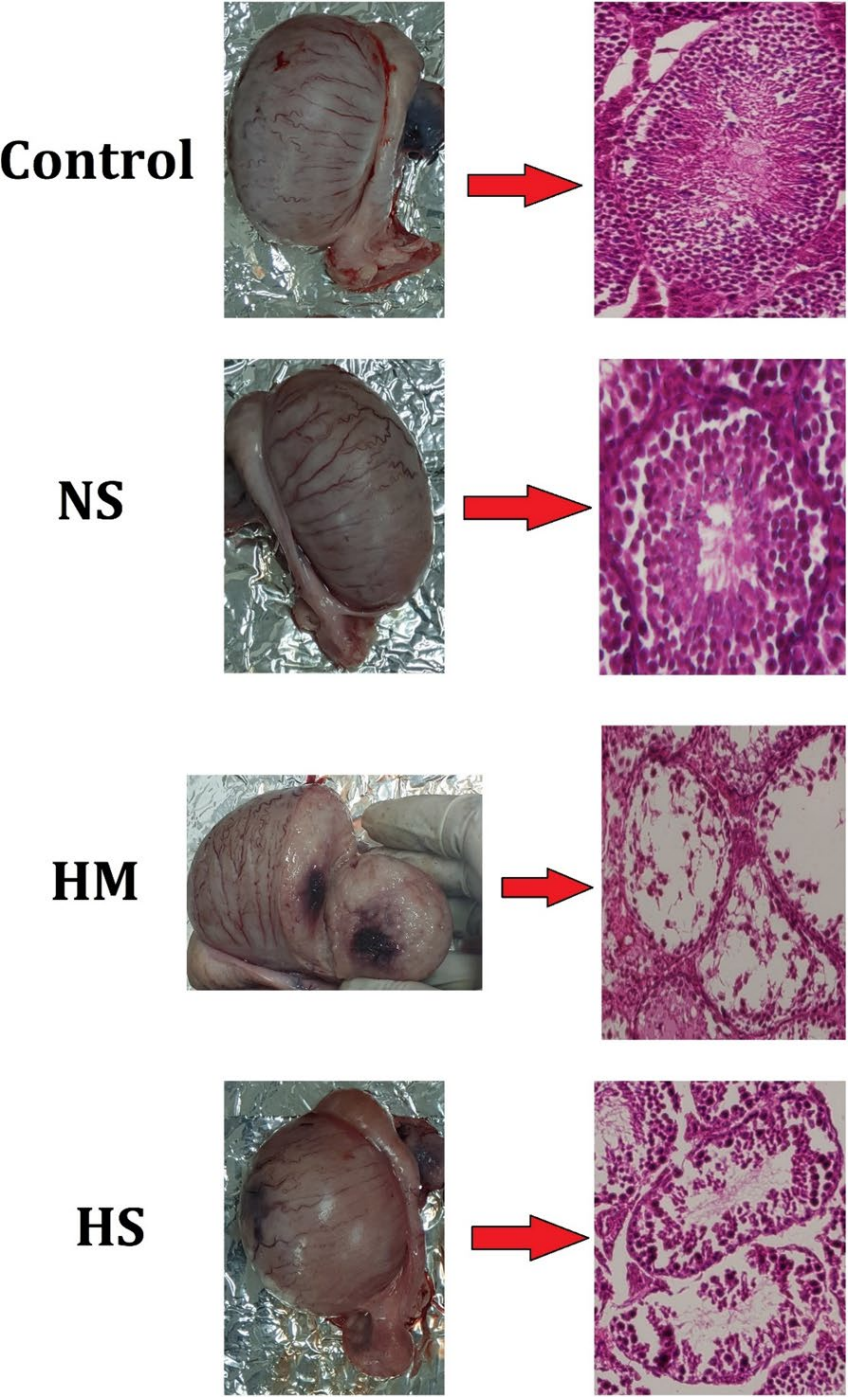
The effects of different treatments on testicular histoarchitecture. Hematoxylin and eosin, 400×). NS: normal saline; HM: hypertonic mannitol; HS: hypertonic saline

Injection of HM and HS induced deletion of germ cells during spermatogenesis, resulting in a dramatic decrease in SCI. Due to the deletion of germ cells, the number of recolonizations and seminiferous tubule diameters were greatly reduced in the HM and HS groups compared to the other groups (*p* ≤ 0.05, Table 7). Injection of HM and HS also resulted in a significant reduction in miotic index. However, the results showed that there was no significant difference between the control group and the normal saline group in these parameters (*p* > 0.05; Table 7). Furthermore, analysis of histological parameters showed that testicular biopsy score, spermiogenesis index, Leydig nuclear diameter, Sertoli nuclear diameter, tubular differentiation index and spermiation index were reduced by injection of HM and HS compared to the other groups (*p* ≤ 0.05, Table 7; Fig. 6).

**Table 7:** Influence of intratesticular injections on histological parameters of donkey testicles in different experimental groups. Values are expressed as mean ± SEM

**Groups**	**Control**	**NS**	**HM**	**HS**
**Analysis**				
**Scrotal circumference (cm)**	24.30 ± 1.45^a^	24.00 ± 1.62^a^	16.18 ± 1.54^b^	15.60 ± 1.26^b^
**Testicular length (cm)**	8.45 ± 0.90^a^	8.09 ± 0.85^a^	5.25 ± 0.93^b^	4.78 ± 0.89^b^
**Testicular width (cm)**	6.41 ± 0.48^a^	6.35 ± 0.41^a^	4.10 ± 0.95^c^	5.19 ± 0.81^b^
**Testicular height (cm)**	6.73 ± 0.71^a^	6.90 ± 0.37^a^	4.46 ± 0.74^c^	5.54 ± 0.96^b^
**Testicular volume (cm**^**3**^**)**	224.41 ± 25.64^a^	220.52 ± 31.19^a^	59.21 ± 8.13^c^	68.12 ± 11.40^b^
**TBS (Johnsen’s score)**	9.13 ± 0.24^a^	8.70 ± 0.46^a^	3.85 ± 0.21^b^	3.42 ± 0.44^b^
**STD (µm)**	269.41 ± 24.30^a^	248.52 ± 39.95^a^	65.96 ± 17.43^c^	71.45 ± 19.78^b^
**Sertoli cell index (SCI)**	24.75 ± 1.25^a^	24.83 ± 1.20^a^	4.21 ± 0.58^b^	4.78 ± 0.34^b^
**Repopulation index (RI)**	91.47 ± 3.18^a^	88.64 ± 3.51^a^	17.85 ± 1.62^b^	16.09 ± 0.67^b^
**Miotic index (MI)**	2.43 ± 0.31^a^	2.51 ± 0.19^a^	0.84 ± 0.25^b^	0.71 ± 0.49^b^
**LCND (µm)**	7.28 ± 0.32^a^	6.81 ± 0.45^a^	2.79 ± 0.17^b^	3.15 ± 0.22^b^
**SCND (µm)**	8.74 ± 0.27^a^	8.30 ± 0.67^a^	5.26 ± 0.73^c^	4.79 ± 0.31^b^
**TDI (%)**	92.43 ± 3.91^a^	92.07 ± 2.84^a^	16.23 ± 0.84^b^	18.11 ± 1.08^b^
**SPI (%)**	94.82 ± 2.48^a^	92.84 ± 2.62^a^	40.56 ± 1.55^c^	45.70 ± 1.42^b^

*NS* normal saline, *HM* hypertonic mannitol, *HS* hypertonic saline, *TBS* Testicular biopsy score, *STD* Seminiferous tubule diameter, *LCND* Leydig nuclear diameter, *SCND* Sertoli nuclear diameter, *TDI* Tubule Differentiation Index, *SPI* Spermiogenesis index

^a,b,c^Different superscripts within the same row demonstrate significant differences (*p* ≤ 0.05)

#### *Bax*, *Caspase-1*, *GSDMD* and *Bcl-2* Gene expressions in testes

**Fig. 7:**
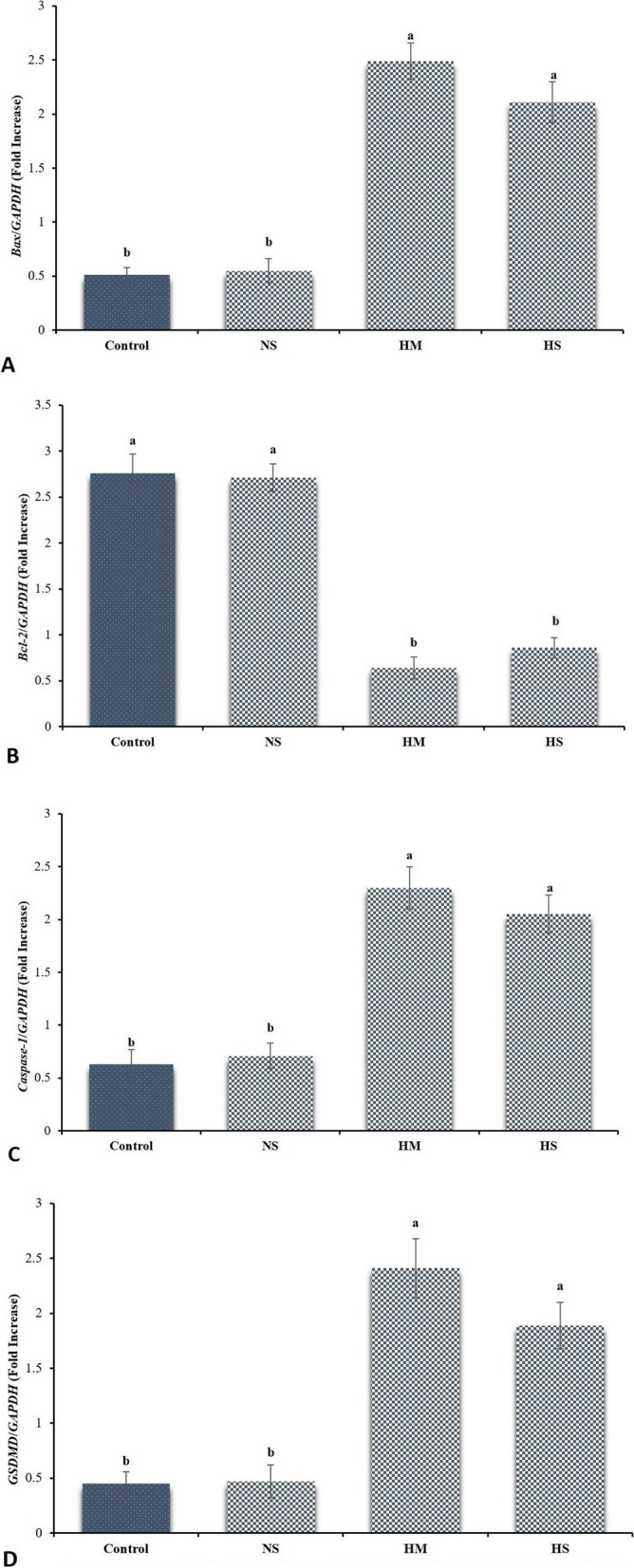
The mRNA levels of (**A**) *Bax*, (**B**) *Bcl-2*, (**C**) *Caspase-1*, and (**D**) *GSDMD* were assessed using semiquantitative reverse transcription-polymerase chain reaction (RT-PCR). The density of *Bax*, *Bcl-2*, *Caspase-1*, and *GSDMD* mRNA levels in testicular tissue was measured by densitometry and normalized to the *GAPDH* mRNA expression level. NS: normal saline; HM: hypertonic mannitol; HS: hypertonic saline. ^ab^Significant differences between groups are indicated by different superscripts (*p* ≤ 0.05; Mean ± S.E.M.)

The *Bcl-2* and *Bax, Caspase-1*, and *GSDMD* mRNA levels were assessed by the qRT-PCR technique. No statistically significant differences were revealed in the *Bcl-2* and *Bax*, *Caspase-1*, and *GSDMD* mRNA levels, between control and normal saline groups (*p* > 0.05; Fig. 7). However, the HM and HS groups showed increases in *Bax*, *Caspase-1* and *GSDMD* mRNA levels compared to the control. However, *Bcl-2* was reduced in the HM and HS groups (*p* ≤ 0.05, Fig. 7), no statistically significant difference was revealed between control and normal saline groups (*p* > 0.05; Fig. 7).

## Discussion

**Fig. 8:**
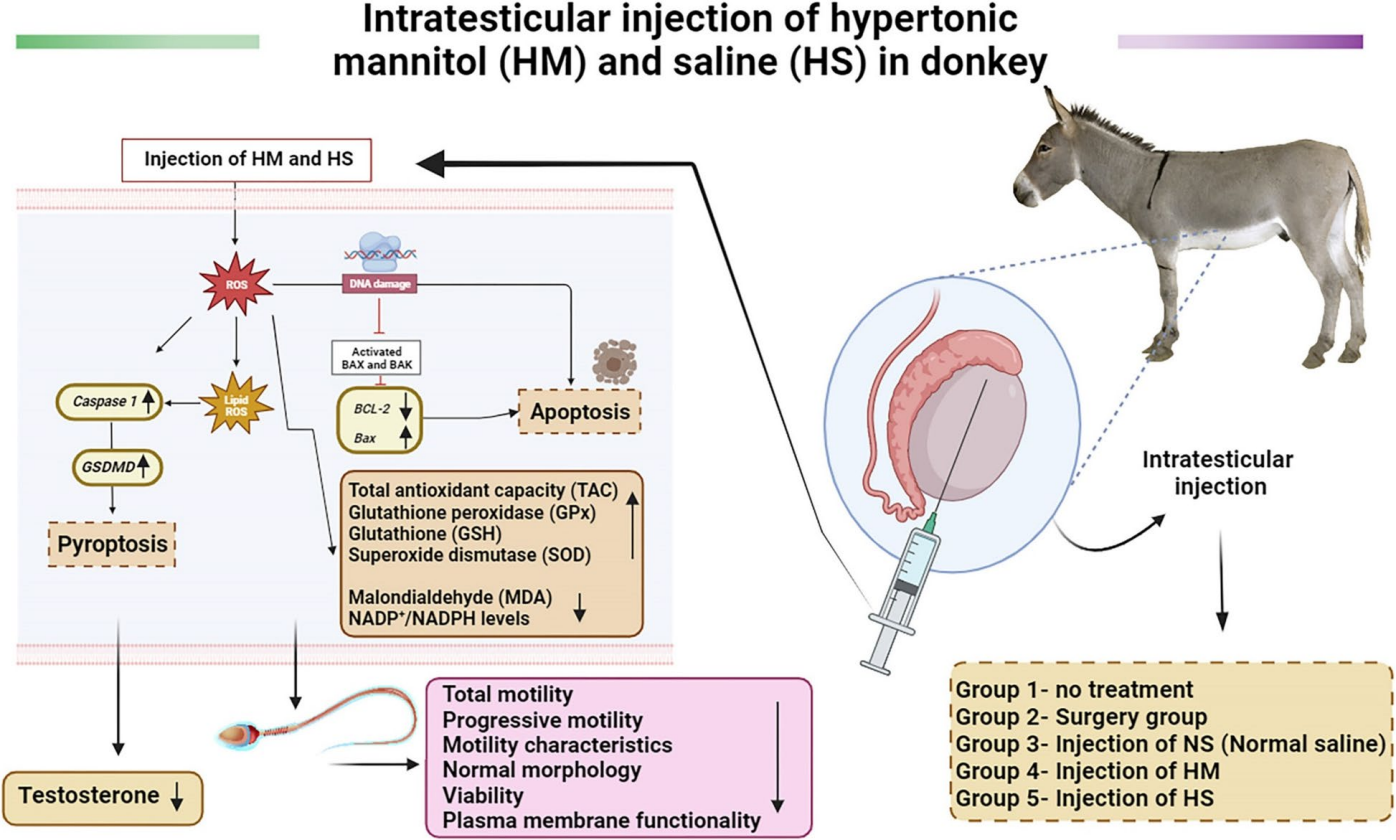
The effectiveness of intravenous injections of mannitol and HTS for chemical sterilization

The effectiveness of intratesticular injections of HM and HS for chemical sterilization was examined and demonstrated in the current study on male donkeys (Fig. 8). In mammals, intratesticular injection of calcium chloride, zinc gluconate, HM, and HS have all been tested for chemical castration [3, 16, 17, 29]. Animals have been reported to be chemically, surgically and mechanically castrated [13, 59, 61]. Because of the risks and disadvantages of surgical castration, we must now turn to non-invasive chemical methods capable of completely stopping spermatogenesis, androgenesis, and libido. These methods must also be effective on a large scale, have a high safety profile and be irreversible after a single treatment [62]. However, it has not yet been possible to find a 100% successful and side effect-free approach to terminating fertility. Each drug or procedure used for this purpose has different advantages and disadvantages [63].

One of the main problems is the adverse side effects promoted by these chemicals in castration [6]. Chemical and drugs like medroxyprogesterone acetate, cyproterone acetate, and LHRH agonists, when used for chemical castration, can cause a notable decrease not just in testosterone levels but also in estradiol. Estrogens are important for men as well, as they have positive effects on skeletal growth, bone maturation, brain function, and cardiovascular health [64]. Side effects of chemical castration include osteoporosis, cardiovascular disease, and impaired glucose and lipid metabolism [65]. Anemia, infertility, depression and hot flashes are other possible consequences. The side effects of chemical castration may worsen over time, as the minimum treatment duration for severe paraphilia with a high risk of sexual violence is three to five years [66]. Our study found no observed side effects in the animals after intratesticular HM and HS in donkey testis. Similar to our finding, a previous study previous study found that side effects such as diarrhea, lethargy, vomiting, scrotal ulceration, and dermatitis were not observed following intratesticular HM injection [29].

Our research found that the HM and HS groups had lower testosterone levels than the other groups. Qualitative testicular section analysis, showing fibrosis in the interstitial space of seminiferous tubules and degeneration of Sertoli and Leydig cells, supported the low testosterone levels in the HM and HS groups in this case. In findings of Maadi et al. (2021) consistent with our findings, injections of HM and HS have been shown to decrease testosterone levels in male rats [29]. Decreased testosterone levels in our study, was similar to studies by other researchers on other animals such as dogs [70, 67] and donkey [3]. In addition, more recently, a single intratesticular injection of HS significantly decreased testosterone levels, simulating the effects of surgical castration in rats and demonstrating the condition of chemical castration [71, 72].

In the present study, administration of HM and HTS had a significant impact on seminiferous tubules, which could be a cause of deterioration in sperm quality. This was reflected in a significant reduction in sperm motility. Sperm motility is commonly used as a measure of testicular toxicity induced by chemicals [73]. The motility of the sperm is also an important characteristic for assessing the fertility of sperm [74]. Progressive motility is the ability of sperm to move straight ahead in a specific direction, which is necessary for sperm migration in the female reproductive system. Intratesticular injection of HM and HTS in animals of the present study significantly decreased total and progressive motility as well as characteristic motility of sperm from the cauda epididymis. Sperm motility is dependent on various metabolic processes and regulatory systems. Two important mechanisms that regulate sperm motility are the calcium (Ca2+) pathway and the cyclic adenosine monophosphate (cAMP) pathway-dependent protein kinase or protein kinase A [38, 40, 75]. Testicular injury can disrupt the normal functioning of the calcium signaling pathway, which is essential for sperm motility and fertilization. This disruption can lead to decreased sperm motility and fertility [76]. Thus, due to the potential damage caused by HM and HTS in the testis, they may lead to a reduction in sperm motility by interfering with the calcium pathways [38, 40, 75, 77]. In addition to gene mutations, sperm abnormalities can also lead to changes in sperm motility [78–80].

The current study showed that administration of HM and HTS by intratesticular injection in donkeys resulted in a significant decrease in sperm viability, PMF and normal morphology. The injection of HM and HTS into the testicles led to damage of the testicular tissue. The oxygen supply to the testicles is rather limited compared to other organs. However, an undisturbed testicle can adjust to this condition and allow proper metabolic function in the seminiferous tubules [81]. Administration of HM and HTS led to a reduction in sperm concentration in the cauda epididymis. The phenomenon observed could possibly be due to breakdown of the seminal epithelium, shrinkage of the testicular parenchyma and a subsequent decline in spermatogenesis. Since the cauda epididymis serves as a site of sperm storage, the observed reduction in epididymal sperm concentration can be interpreted as an indication of reduced sperm production in the testes. A decrease in sperm count in animals has been found to be associated with subfertility or infertility [82]. Several studies have shown that sperm survival and PMF integrity are negatively affected by the presence of various chemicals that disrupt membrane integrity resulting in higher ab [61, 79, 83, 84]. The present study revealed a striking disparity in sperm morphology between the control and treatment cohorts. The present study revealed several sperm abnormalities including coiled tails, curved necks, double tails, double-tailed macrocephals, microcephals, elongated heads and hairpin tails [85]. According to Kwak et al. (2013) it has been shown that administration of sodium chloride injection can result in significant degenerative changes and extensive infiltration of immune cells into the seminiferous tubules of the testicles [16].

Male infertility can be linked to several factors such as genetic abnormalities, hormonal imbalances, problems in spermatogenesis, poor sperm quality and sperm DNA fragmentation [86]. According to a study by Kumaresan et al. (2017) there was a remarkable association between DNA damage and fertility in dairy bull [1]. Inbull. Similar reports of the role of sperm DNA integrity in fertility and/or semen quality exist for other livestock including stallions, boars and rams [87–89]. A study by Gromadzka-Ostrowska et al. (2012) found that even small amounts of 20 nm AgNPs administered to the bloodstream have a toxic effect on germ cells, as evidenced by the reduced number of spermatozoa and significantly higher levels of DNA damage in germ cells, which can ultimately lead to reduced reproductive potential of the organism [90]. In our study, acridine orange staining results showed that DNA damage was increased HM and HS groups compared to the control group suggesting that injection of HM and HS could have a negative effect on DNA. Similar to our funding, a study showed that injection of HM and HTS could increase sperm DNA damage in male rats [29]. The study by Zeini et al. (2010) demonstrated that incomplete and defective spermatogenesis can lead to fragmentation of DNA strands. On the other hand, our study showed increased DNA damage after the injection of HM and HTS in the testes might be due to the increase in apoptosis in the testes due to the injection of these substances. In support of our study, a study showed that DNA damage due to apoptosis occurred mainly in the testes during spermatogenesis [92].

The susceptibility of testicular tissue to oxidation is attributed to the increased presence of polyunsaturated fatty acids in sperm membranes [93]. This study showed that administration of HM and HS in donkeys resulted in a significant induction of oxidative stress. The results of this study showed that there was a large increase in NADP+/NADPH and MDA levels in testicular tissue in both the HM and HS groups. In addition, there was a significant reduction in TAC, GPx, SOD, and GSH levels in these groups. Our study confirmed the findings of Abou-Khalil et al. (2020), who showed that chemical castration resulted in a reduction in TAC in male donkeys [4]. Oxidative stress has been identified as a potential toxicity mechanism for HM and HTS. When the concentration of ROS in the testicular tissue increases, the lipids of the plasma membrane are damaged and peroxidized leading to the destruction of intracellular components [94]. The increase in the formation of free radicals in the testicles due to administration of HM and HTS is further confirmed by the decrease in the activities of the testicular GPx, SOD and GST enzymes, as these enzymes are considered key free radical scavengers in the male reproductive organs [7]. According to Asghari et al. (2016), GPX is an important enzyme in the male reproductive system that plays a crucial role in reducing free radicals [94].

This study examined the histological and morphometric indicators of testicular tissue to assess the effects of intratesticular injection of HM and HTS. The results showed significant changes in these indicators similar to the observed seminiferous tubule atrophy. Histopathological studies of the epididymis of donkeys treated with HM and HTS revealed a marked reduction in the presence of spermatozoa in the epididymal lumen. The potential reduction in sperm count can be attributed to several factors including testicular degeneration, testicular parenchymal atrophy, seminal epithelial degeneration and poor spermatogenesis. These results were consistent with previous research in this area. The study by Maadi et al. (2021) showed that administration of HM and HTS in rats resulted in testicular atrophy [29]. Previous studies have documented comparable results in terms of impaired spermatogenesis and reduced sperm parameters in dogs treated with sodium chloride solution [28]. According to Oguejiofor et al. (2020) the observed decrease in epididymal sperm concentration can be attributed to a reduction in spermatogenesis in the testes [95].

Emir et al. (2008) conducted a study in which hypertonic saline was injected bilaterally into the testicles of rats [71]. The results of the study indicated the presence of coagulative necrosis in all testes. The study by Kwak et al. (1993) demonstrated the presence of significant degenerative changes in the seminiferous tubules as well as significant infiltration of immune cells in the hypertonic saline treated group [26]. The vacuolation observed in germ cells within the seminiferous tubules is the result of degenerative changes or the physical exhaustion of the germ cells. This phenomenon serves as an early morphological sign of possible Sertoli cell disorders [96, 97]. In addition, it can also indicate fluid imbalances originating from Sertoli cells [98]. The histological manifestation of testicular cell atrophy is characterized by the partial or complete absence of mature spermatids in the tubular lumen. With increasing atrophy, there is a constant loss of cells in the deeper layers. The presence of a limited number of spermatogonia cells and Sertoli cells served as a clue [99]. Our study, demonstrated that administration of HM and HTS resulted in the occurrence of vacuolation in the germ cells of the seminal tube [100]. 

It is believed that changes in certain physicochemical states have the potential to induce tissue necrosis. The study by Goslvez et al. (2015) showed that the presence of apoptotic markers such as *Fas*, *Bcl-X*, *p53* and *Annexin V* in both testicular and mature sperm provided evidence for the involvement of apoptosis in DNA break formation [92]. However, the relationship between the presence of these typical markers of apoptosis in spermatozoa and testes and the degree of DNA fragmentation was not clear [101]. Abortive apoptosis is a phenomenon in which the defective sperm cell escapes programmed cell death and is present in the ejaculate. However, these defective sperm with partial DNA breaks retain their fertilization potential but are unable to support pregnancy, resulting in early embryo loss [102]. Apoptosis is an important factor in the mammalian evolutionary process and acts as a quality control mechanism responsible for the elimination of cells that are damaged, defective, aberrant or located in inappropriate regions [103]. Previous studies have shown that extended sperm viability depends on maintaining the balance between apoptotic and anti-apoptotic genes [26]. The aim of this work was to investigate the involvement of *Bax* transcription factors in DNA damage caused by HM and HTS. For this purpose, RNA expression profiles were analyzed. The study results indicated that the intratesticular injection of HM and HS led to a significant increase in *Bax* gene expression and a simultaneous decrease in *Bcl-2* gene expression. According to Ozen et al. (2008) *Bcl-2* is a protein with anti-apoptotic properties. Its function is to inhibit the activation of caspase proteins, thus delaying the onset of apoptosis [104]. This is achieved by impeding the entry of the *Bax* protein into the mitochondrial membrane and subsequent release of cytochrome c [105]. Several studies have demonstrated the ability of free radicals to phosphorylate and alter *Bcl-2* proteins, thus, serving as indicators of apoptosis [106]. In our study, it was found that increased expression of the *Bax* gene facilitated the induction of apoptosis and cell death by HM and HTS. In a previous study by Malek et al. (1998) it has been shown that endothelial cells undergo apoptosis when exposed to mannitol [107]. A study by Zhang et al. (1999) have shown that mannitol induces apoptosis and mortality of renal tubular epithelial cells in vitro by inducing oxidative stress and disrupting the cytoskeleton [108]. Due to the lack of effective protective mechanisms in the testis against severe oxidative damage, oxidative stress induced by HM and HTS in testicular tissue could result in testicular tissue destruction, seminiferous tubule germ cell death and azoospermia that eventually led to infertility [109].

Overall, these results showed that injection of mannitol and HTS increased expression of the pyroptosis and apoptosis genes and decreased expression of the anti-apoptotic gene suggesting that it could be the process of apoptosis and pyroptosis in the testis.

Activation of *GSDMS* family proteins is necessary for the recently identified type of programmed cell death known as pyroptosis [110]. Pyroptosis in contrast to apoptosis, is mainly characterized by DNA damage, plasma membrane rupture and release of intracellular pro-inflammatory chemicals [111]. Cell membrane rupture, increased expression of essential pyroptosis proteins such as *NLRP3* and *caspase-1*, and release of inflammatory molecules such as L-1B and L-18 are all hallmarks of the particular type of inflammatory cell death known as pyroptosis. The traditional mechanism of pyroptosis is *caspase-1*-mediated and caspase-1 activation is dependent on the *NLRP3* inflammasome [112]. Recent researches [113, 114] have shown higher expression of *NLRP3* in a number of testicular diseases. Also, the *GSDMD* gene plays a critical role in the pyroptosis process, which is a form of programmed cell death that is mediated by inflammatory caspases. When activated, *GSDMD* forms pores in the cell membrane, leading to cell swelling and rupture, and the release of pro-inflammatory cytokines. This process is important for the immune response to infection and inflammation [115]. In our study, by examining the expression of pyroptosis, including testicular tissue, it was shown that intratesticular injection of HM and HTS could increase expression of *caspase-1* and *GSDMD* genes. Similar to our foundlings, Zhou et al. (2020) discovered that an increase in ROS can also cause pyroptosis [116]. According to the several studies [117–119], cadmium exposure leads to pyroptosis of neuronal, lymphocyte and vascular endothelial cells via activation of the *NLRP3*-inflammasome complex. Due to intratesticular injection of HM and HTS, ROS may have grown rapidly and significantly in a short period of time, causing DNA breakage in the testicular tissue to increase beyond the body’s ability to repair the damage. In addition, our study found that the testis had high levels of *GSDMD* expression, which damaged Leydig cells and could be the main factor behind the decrease in testosterone levels. In addition, Consistent with the present study, Zhang et al. (2021) showed that cadmium and molybdenum could enhance the expression of *GSDMD* and *GSDME* in renal tubular epithelial cells leading to pyroptosis of the epithelial cells [120]. In the testicular tissue of mice treated with cadmium, Zhou et al. (2022) also discovered higher expression of *GSDMD* and *GSDME* [121].

## Conclusion

It could be concluded that exposure to HM and HS induced activation of the inflammasome by increasing ROS levels and also further stimulated pyroptosis and apoptosis in testicular tissue of donkeys with expression genes. The results of the current study further showed that HM and HS induced oxidative stress by disrupting the osmotic balance in testicular cells. HS and HM as toxic biochemical stressors impaired androgen access and energy metabolism, damaged sperm DNA, caused functional damage to the plasma membrane, increased expression of apoptotic and pyroptotic genes and could poison the reproductive system of the male donkeys. The intratesticular injection of these substances could in future be a risk-free postoperative substitute for surgical castration in the sterilization of donkeys. However, further investigation is needed to determine the effects of intratesticular HM and HS on in vivo or in vitro fertility.

## Data Availability

This article contains all the data that were created or evaluated during the research.

## Abbreviations

HM: Hypertonic mannitol
HS: Hypertonic saline
NS: Normal saline
TM: Total motility
PM: Progressive motility
VCL: Curvilinear velocity
VSL: Straight-line velocity
VAP: Average path velocity
STR: Straightness
LIN: Linearity
ALH: Amplitude of lateral head displacement
BCF: Beat-cross frequency
PMF: Plasma membrane functionality
TAC: Total antioxidant capacity
MDA: Malondialdehyde
CAT: Catalase
GPx: Glutathione peroxidase
SOD: Superoxide dismutase
GSH: Glutathione
AO: Acridine orange
ROS: Reactive oxygen species
DAMPs: Danger-associated molecular patterns
ASC: Apoptosis-associated Speck-like protein containing a CARD
NaCl: Sodium chloride
